# Abrogation of atypical neurogenesis and vascular-derived EphA4 prevents repeated mild TBI-induced learning and memory impairments

**DOI:** 10.1038/s41598-020-72380-1

**Published:** 2020-09-21

**Authors:** Kisha Greer, Erwin Kristobal Gudenschwager Basso, Colin Kelly, Alison Cash, Elizabeth Kowalski, Steven Cerna, Collin Tanchanco Ocampo, Xia Wang, Michelle H. Theus

**Affiliations:** 1 Graduate Program in Translational Biology, Medicine, and Health, Virginia Tech, Blacksburg, VA 24061 USA; 2 The Department of Biomedical Sciences and Pathobiology, Virginia Tech, Blacksburg, VA 24061 USA; 3 Center for Regenerative Medicine, Virginia-Maryland College of Veterinary Medicine, Blacksburg, VA 24061 USA; 4 Center for Engineered Health, Virginia Tech, Blacksburg, VA 24061 USA

**Keywords:** Neuroscience, Neurogenesis

## Abstract

Brain injury resulting from repeated mild traumatic insult is associated with cognitive dysfunction and other chronic co-morbidities. The current study tested the effects of aberrant neurogenesis in a mouse model of repeated mild traumatic brain injury (rmTBI). Using Barnes Maze analysis, we found a significant reduction in spatial learning and memory at 24 days post-rmTBI compared to repeated sham (rSham) injury. Cell fate analysis showed a greater number of BrdU-labeled cells which co-expressed Prox-1 in the DG of rmTBI-injured mice which coincided with enhanced cFos expression for neuronal activity. We then selectively ablated dividing neural progenitor cells using a 7-day continuous infusion of Ara-C prior to rSham or rmTBI. This resulted in attenuation of cFos and BrdU-labeled cell changes and prevented associated learning and memory deficits. We further showed this phenotype was ameliorated in EphA4f.^/f^/Tie2-Cre knockout compared to EphA4f.^/f^ wild type mice, which coincided with altered mRNA transcript levels of MCP-1, Cx43 and TGFβ. These findings demonstrate that cognitive decline is associated with an increased presence of immature neurons and gene expression changes in the DG following rmTBI. Our data also suggests that vascular EphA4-mediated neurogenic remodeling adversely affects learning and memory behavior in response to repeated insult.

## Introduction

Traumatic brain injury (TBI) is a leading cause of disability and death worldwide, with mild TBI comprising more than 70% of TBI-related emergency visits^1^. Importantly, repeated trauma, within the metabolic window of repair, may prolong recovery and increase the risk of developing severe cognitive impairments and neurodegenerative disorders^2–6^. The mechanisms involved in regulating these chronic changes remain ill-defined. Hippocampal neurogenesis, the birth of new neurons, is a fundamental process that occurs throughout adulthood and contributes to learning and memory behavior in mammals^7–9^. Adult neurogenesis in the dentate gyrus (DG) of the hippocampus is highly coordinated and involves the proliferation, differentiation, migration, and survival of neural stem-progenitor cells (NSPCs) and immature neurons. Regulation of homeostasis in this region of the brain relies on environmental cues in the surrounding niche, local secretion of soluble mediators and cell-to-cell contact proteins^10–13^.

Neurogenic ablation studies in mammalians under naïve conditions demonstrate impairments in adult hippocampal-dependent spatial learning and memory, supporting a key role for neurogenesis in cognitive behavior^14,15^. Furthermore, employment of *delta-nestin HSV-TK* mice shows that unilateral, moderate cortical contusion TBI-induced hippocampal remodeling requires activation of quiescent early progenitors^16^. Genetic or drug-mediated suppression of this response has been shown to impair spontaneous cognitive recovery after moderate lateral fluid percussion injury (FPI)^15^. However, additional studies suggest that FPI-induced neurogenesis may adversely affect long-term outcomes and contribute to the development of epileptogenesis^17^. Conflicting evidence also exists regarding the influence of neurogenic ablation on pilocarpine-induced seizures and epilepsy-associated cognitive deficits^18^. Murine models have also established that ischemia-induced aberrant neurogenesis in the hippocampus is associated with cognitive decline^19,20^ which is ameliorated following either pharmacological or genetic neurogenic ablation strategies^21^.

Atypical proliferation and migration of immature neurons to the outer granule layer of the dentate gyrus and hilus occurs in several models of single moderate TBI^22,23^, however, it remains unclear whether these TBI-induced changes are model dependent, benefit recovery from injury or mediate the establishment of an aberrant state in the hippocampal circuitry. Compared to single mild TBI, new findings suggest that repeated mild TBI (rmTBI) in a murine model results in significant chronic learning and memory deficits^24–29^. In the present study, we evaluated whether these effects correlate with substantial neurogenic changes in the DG using BrdU fate labeling, the causal relationship between these events and the potential mechanisms involved using cell-specific knockout mice. Strong evidence supports the role of vascular-derived ephrin type-A receptor 4 (EphA4) in the regulation of hippocampal neurogenesis^30,31^. Our recent findings implicate EphA4 as critical player in cerebrovascular function^32–34^. EphA and EphB class receptors, the largest group of receptor tyrosine kinases, and their membrane bound ephrin ligands mediate cell-to-cell contact signaling involved in numerous cell functions including cell migration, proliferation, survival, axon guidance, synaptogenesis, and vascular remodeling among other roles^35–38^. Using endothelial cell (EC)-specific EphA4 knockout (KO) mice (EphA4f.^/f^/Tie2-Cre) and wild type (WT) mice (EphA4f.^/f^)^32,33^, the current study sought to advance our understanding of Eph signaling in aberrant neurogenesis as it relates to brain trauma. Our findings uncovered the direct involvement of atypical neurogenesis in the observed hippocampal-dependent learning and memory impairments following rmTBI. We further provide evidence that vascular-derived EphA4 receptor tyrosine kinase is a key player in this response.

## Methods

### Animals

All mice were housed in an AAALAC accredited, virus/specific antigen-free facility with a 12 h light–dark cycle; food and water ad libitum. Male CD1 mice were purchased from Charles Rivers and reared until age P60–P90 for experimentation. Female EphA4f.^/f^ and male EphA4f.^/f^/Tie2-Cre^+/−^ on the CD1 background were bred as previously described^32,33^ to generate the appropriate number of male mice, reared until age P60–P90, required for each experiment. All experiments were conducted in accordance with the NIH Guide for the Care and Use of Laboratory Animals and were conducted performed under the approval of the Virginia Tech Institutional Animal Care and Use Committee (IACUC; #17-093) and the Virginia-Maryland Regional College of Veterinary Medicine.

### Repetitive TBI and sham protocol

Adult male mice (P60–90) were anesthetized using ketamine (100 mg/kg) and xylazine (10 mg/kg) subcutaneous injection and positioned in a stereotaxic frame. Body temperature was monitored and maintained at 37 °C with a controlled heating pad set and rectal probe (Harvard Apparatus). Injury was induced by a program controlled cortical impactor (Φ = 5-mm flat tip) connected to an eCCI-6.3 device (Custom Design and Fabrication, LLC) at a velocity of 5.0 m/s, depth of 1.0 mm, and 200 ms impact duration. The flat impactor tip was positioned directly between lambda and bregma. Following injury, the incision was closed using Vetbond tissue adhesive (3 M, St. Paul, MN, USA) and the animals were placed into a heated cage and monitored until fully recovered from anesthesia. Repeated surgeries for TBI and sham were conducted once every 2 days for a total of 5 surgeries^26^.

### BrdU and the Ara-C administration

Bromodeoxyuridine (BrdU; 50 mg/kg/day) was injected on day 0–5 during the repeated injury paradigm^39^. For AraC Infusions, Alzet Mini-Osmotic Pumps Model 1007D (DURECT Corporation, Cupertino, CA) were used to provide continuous systematic delivery of the vehicle saline control or 2% AraC (Sigma, St. Louis, MO, USA) after subcutaneous implantation^40^. Infusions were performed 7 days prior to rmTBI and rSham at which time the pumps were removed on the day of surgery.

### Barnes maze evaluation

Mice were evaluated for learning and memory utilizing the Barnes Maze (92 cm diameter, 20 holes, 5 cm holes) (Maze Engineers, Boston, MA USA) and the Ethovision XT software (Noldus, Leesburg, VA USA) at the same time each day in the early am^26^. Visual cues were provided on the wall above the table and an overhead light was used for illumination and the maze is cleaned to remove residual olfactory cues from previous runs. Mice were placed in the center of the table under an opaque jar for 20 s until the start of the trial. Mice were first pre-trained to enter the escape box by allowing them to freely explore the maze for 5-min total with no anxiety-inducing lights. During this training phase, mice were prompted to enter the hole 3 times, once at the very beginning and once at the very end of the 5-min trial if they had not already entered the escape box. No analyses were collected during the training phase. The following three days (days 21–23 post primary-injury), mice were evaluated for distance, time, and the number of errors to find the escape box each day for 180 s/trial in 3 trials/day for a total of 9 trials. Primary errors were calculated every time the subject poked its head into a hole that was not the target hole. During each of these acquisition trials, if the mice did not enter the escape box, they were prompted to enter and remain for 30 s before returning to their home cage. The average of the 3 daily trials were reported.

### Tissue processing and staining

Mice were euthanized by overdose of isoflurane. Brains were harvested at both 9 and 24 days post-primary injury (dppi) and immediately fresh frozen and embedded in OCT. Serial coronal sections were processed on a cryostat (Cryostar, Fisher Scientific) using five 30 μm coronal sections spaced ten sections apart and placed in the − 80 °C freezer as previously performed^32,41–43^. Sections were fixed with 10% buffered formalin (Fisher Scientific) then washed with 1X phosphate-buffered saline (PBS) and blocked for 1 h in 2% fish gelatin/0.2%Triton. For BrdU staining, sections were placed in 2 N HCL/0.2%Triton solution and placed in a 37 °C incubator for 45 min, then transferred into 0.1 M sodium borate buffer for 10 m, washed with 1X PBS then blocked in 2% fish gelatin/0.2%Triton (Sigma Aldrich) for 1 h at room temperature (RT). Primary antibodies, (Table 1), were applied blocking solution and incubated overnight in the 4 °C. The following day, sections were washed with 1X PBS then secondary antibodies were added (1:250 donkey anti-rabbit Alexa-Fluor 488 and 1:500 donkey anti-rat Alexa-Fluor 594). The sections were washed with 1X PBS before mounting in DAPI-G Fluoromount (Southern Biotech).

### Stereological analysis

Total Cell counts for the granular cell layers (GCL) and hilus of the dentate gyrus (DG) (mm^3^) were assessed by a blinded investigator using Optical Fractionator probe from MBF StereoInvestigator software (MicroBrightField, Williston, VT, USA) and an upright Olympus BX51TRF motorized microscope (Olympus America, Center Valley, PA, USA). Each contour included the entire right and left dentate gyrus structures. Grid size was set at 150 × 150 mm with a 75 × 75 mm counting frame, counting was performed used exhaustive analysis. Four (time course study) to five (AraC study) serial coronal sections were used to analyze both hemispheres of the brain. Total estimated number for each hemisphere was combined during data analysis in the MBF program, graphs are represented as estimated total number.

### Quantitative real-time PCR

Total RNA from the microdissected dentate gyrus was isolated according to manufactures instructions using TRIzol reagent (Ambion). Quantification of RNA was done using spectrophotometer ND-1000 (NanoDrop). We then used 1 µg of total RNA for reverse transcription reaction using iScript cDNA synthesis kit (Biorad, Hercules, CA). Next qRT-PCR analysis, used 50 ng cDNA per reaction and was amplified using iTaq Universal SYBR Green Supermix (Biorad, Hercules, CA). We calculated expression changes using ΔCq values relative to the internal control *gapdh* for each sample. Data was represented as relative expression, which was calculated by normalizing each value to the average WT sham for each gene. All primers (Table 2) showed a primer efficiency range from 87 to 113%.

### Statistical analysis

GraphPad Prism, version 7 (GraphPad Software, Inc., San Diego, CA) was used to graph all data. Multiple comparisons were done using repeated measures two-way ANOVA for behavior or one-way ANOVA for stereology where appropriate followed by Bonferroni post-hoc test for multiple pairwise examinations. Significant changes were identified if *p* was less than 0.05. Mean values are represented together with the standard error of mean (SEM). Sample size was determined based on G*Power 3 (Universitat Dusseldorf, Germany) to retrieve sample size with an acceptable power range between 80–90%. All animals were coded and a double-blinded strategy was used for statistical analysis.

## Results

### Repeated mild TBI (rmTBI) induces learning and memory deficits and enhanced neurogenesis

**Figure 1:**
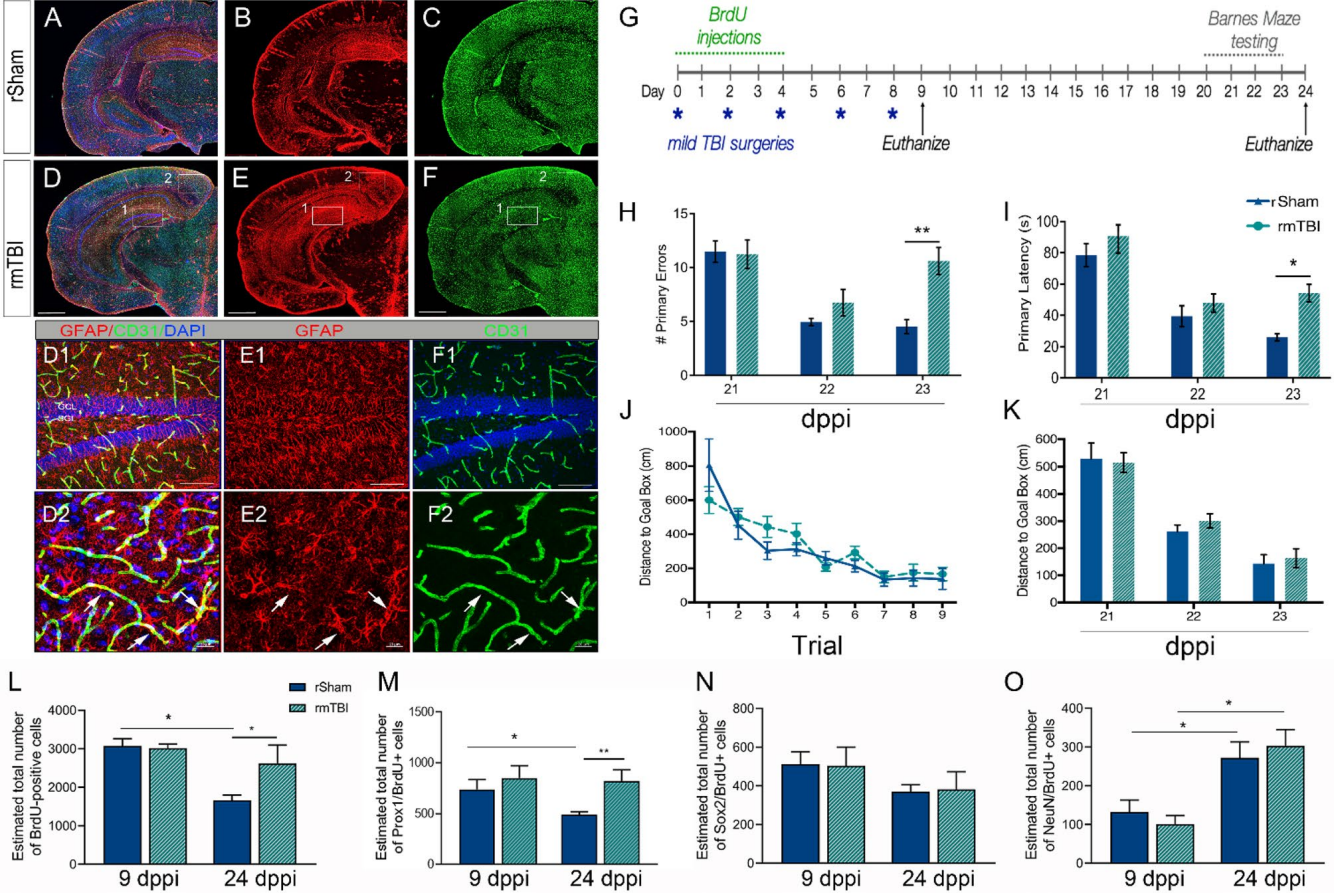
rmTBI induces GFAP immunoreactivity, learning and memory impairments, and expansion of neuroblast/immature neurons. (**A**–**C**) Representative maximum projection z-stack confocal mosaic image of serial section stained for GFAP (red), CD31 (green) and DAPI (blue) in rSham at 24 dppi. (**D**–**E**) Representative confocal image at 24 dppi rmTBI showing increased GFAP immunoreactivity in the hippocampus (**D1**–**F1**; insert) and cortex (**D2**–**F2**; insert). (**G**) Experimental timeline showing repeated mild TBI surgeries (*blue), daily BrdU injections (green), behavioral testing (grey), and two time-points for tissue collection (arrows, black). The rmTBI mice show an increase in the # primary errors (**H**) and latency in seconds (s) (**I**) made during Barnes Maze testing at 23 dppi. No difference was found in the distance to goal box per trial (**J**) or day (**K**). The number of BrdU (**L**) and Prox1/BrdU-positive (**M**) cells in the DG was increased in rmTBI mice while no change was seen in Sox/BrdU (**N**) or NeuN/BrdU (**O**) at 24 dppi compared to rSham. Scale = 1 mm in (**A**–**F)**; 500 µm in (**D1**–**F1**) and 20 µm in (**D2**–**F2**). Data are represented as mean ± SEM. Two-way ANOVA for repeated measures with Bonferroni post-hoc. *p < 0.05, and **p < 0.01. n = 10–15 mice per group for behavior and n = 7–12 for stereology.

To investigate the contribution of neurogenesis to learning and memory deficits following rmTBI, we utilized a previously established mouse model^26^. We subjected mice to five mild closed head impacts, spaced 2 days apart followed by Barnes Maze analysis at 21–23 days post-primary injury (dppi) and histological testing using bromodeoxyuridine (BrdU) labeling at 9 and 24 dppi (Fig. 1G). We observed changes in expression of GFAP immunoreactivity in the cortex, entorhinal cortex, and hippocampus of rmTBI mice (Fig. 1D–F) as compared to repeated sham-injured (rSham) mice (Fig. 1A–C). We also found prominent GFAP immunoreactivity on CD31-positive vessel in the dentate gyrus (DG) at 24 dppi rmTBI (Fig. 1D1–F1) and cortex (Fig. 1D2–F2). In addition, we developed a new, shortened protocol to test for learning and memory deficits using the Barnes Maze (Fig. 1G). Using this method, rmTBI mice displayed a significant increase in the number of primary errors (Fig. 1H) (F_(1,22)_ = 4.43; *p* = 0.04) and latency (Fig. 1I) (F_(1,22)_ = 5.45; *p* = 0.03) compared to rSham at 23 dppi. No difference was found in the distance traveled to the goal box by trial or day (Fig. 1J, K) (F_(1,39)_ = 0.24; *p* = 0.62). These findings confirm previous studies that show enhanced GFAP immunoreactivity as well as learning and memory deficits following rmTBI^26^. Next, BrdU was injected at 0–4 dppi to label and track the fate of neural stem/progenitor cells (NSPCs) in the DG quantified by using non-biased stereology. At 24 dppi post-rSham, we found a significant reduction in the number of BrdU-positive cells in the DG compared to 9 dppi rSham (Fig. 1L). The observed reduction of BrdU-labeled cells over time in rSham mice was prevented in rmTBI mice (F_(1,22)_ = 5.133; *p* = 0.03). The population of BrdU-labeled cells that remained in the rmTBI DG displayed double-labeling with Prox1 (Fig. 1M), a marker for proliferating neuroblasts and immature neurons^44^, but not NSPC marker Sox2^45^ (Fig. 1N) or mature neuronal marker NeuN^46^ (Fig. 1O). These data demonstrate that learning and memory impairments correlate with an atypical increase in the number of immature neurons in the DG.

### Aberrant neurogenesis plays a role in learning and memory deficits following rmTBI

**Figure 2:**
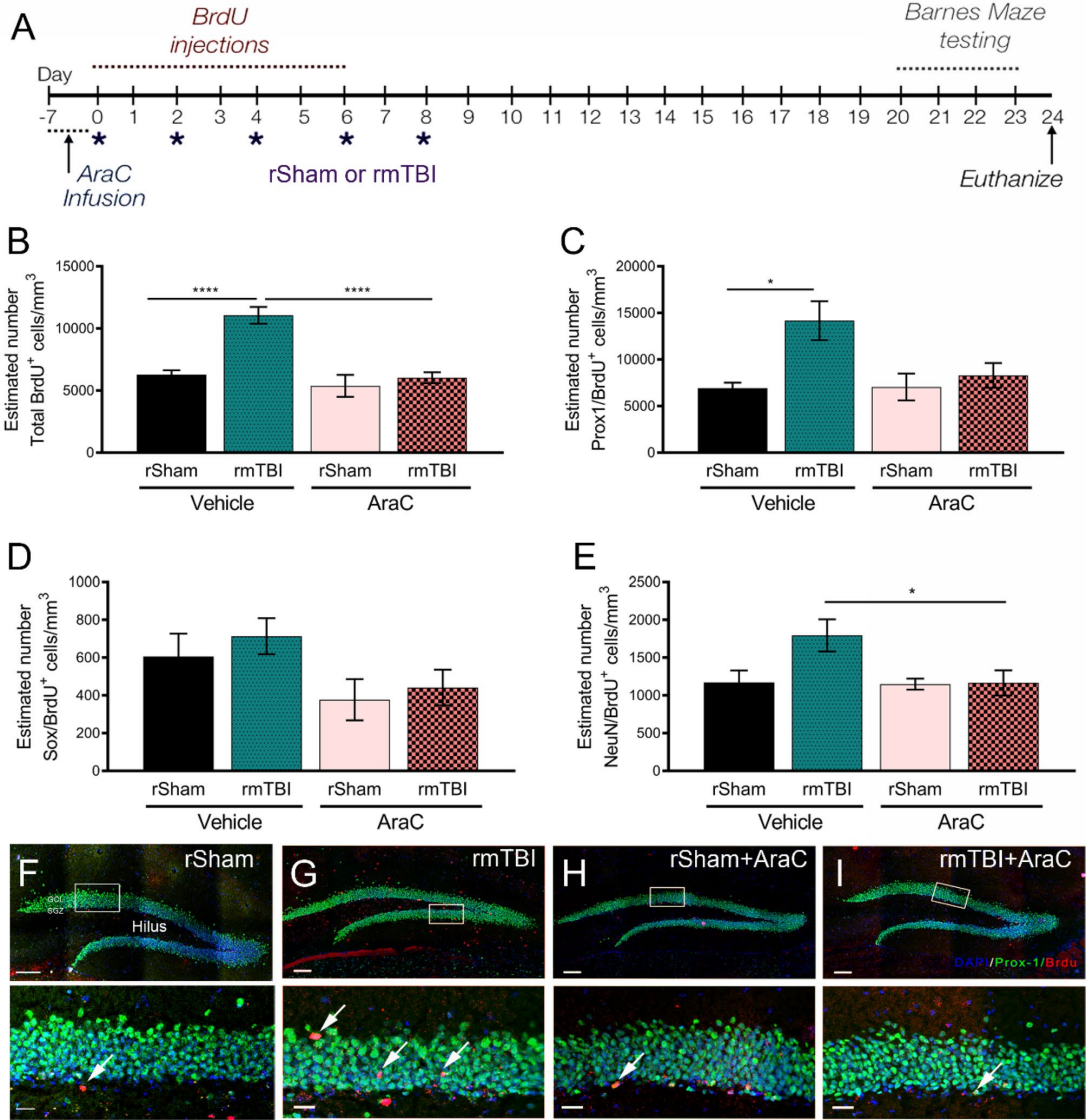
AraC rescues rmTBI-induced aberrant neurogenesis. (**A**) Experimental time line showing 7 days pre-infusion of vehicle or 2% AraC prior to rSham or rmTBI, 50 mg/kg/day BrdU injections, repeated injuries and behavior testing paradigm. (**B**) Quantified data showing estimated number of BrdU+ cells/mm^3^ by non-biased stereology in the DG at 24 dppi. A significant attenuation of increased BrdU-labeling was seen in AraC-treated rmTBI mice compared to vehicle rmTBI. (**C**) AraC also reduced the number of Prox1/BrdU double-positive cells compared to vehicle rmTBI. No significant differences in the estimated number of Sox2/BrdU or NeuN/BrdU positive cells were seen between groups. (**F**–**I**) Representative confocal maximum projection z-stack images of the DG immunolabeled with BrdU (red), Prox1 (green) and DAPI (blue) with inset (white boxes). Scale = 250 µm in (**F**–**I**) and 50 µm in (**F**–**I**) insets. Data are represented as mean ± SEM. One-way ANOVA with Bonferroni post-hoc. *p < 0.05, ****p < 0.000. n = 6–10 mice.

We evaluated whether the atypical changes in hippocampal neurogenesis contribute to cognitive dysfunction as a result of rmTBI. To test this, we pharmacologically suppressed neurogenesis by delivering cytosine arabinoside (AraC) or vehicle control^15,47^ using subcutaneous implants of mini-osmotic pumps and daily i.p. injections of 50 mg/kg BrdU for 7 days prior to rmTBI or rSham (Fig. 2A). After 7-days infusion (0 dppi), we show AraC suppressed BrdU immunolabeling in the DG compared to vehicle-infused mice (Supp. Figure 1). Using this strategy, we then performed rmTBI or rSham following removal of AraC or vehicle osmotic pumps and evaluated histological and behavior changes. Similar to our initial findings, we observed vehicle-treated rmTBI mice showed a significant increase in the number of BrdU-positive cells in the DG compared to vehicle-treated rSham at 24 dppi (Fig. 2B). This effect was attenuated in rmTBI mice subjected to neurogenic suppression prior to the onset of repeated injury (F_(3,28)_ = 4.43; *p* = < 0.001). We also found that Ara-C-treated rmTBI mice showed a trend toward rescue of enhanced Prox-1+/BrdU+ cell numbers seen in the vehicle rmTBI DG (Fig. 2C, F–I) (F_(3,24)_ = 4.35; *p* = 0.01). No differences were found in the number of Sox+/BrdU+ (Fig. 2D) (F_(3,27)_ = 2.11; *p* = 0.12) double-labeled cells between vehicle or AraC-infused rmTBI or rSham mice, however, the number of NeuN+/BrdU+ cells is statistically significant only between vehicle and Ara-C rmTBI mice (Fig. 2E) (F_(3,26)_ = 4.37; *p* = 0.01). This demonstrates that early suppression of neurogenesis can prevent rmTBI-induced expansion of the Prox-1-positive population in the DG.

**Figure 3:**
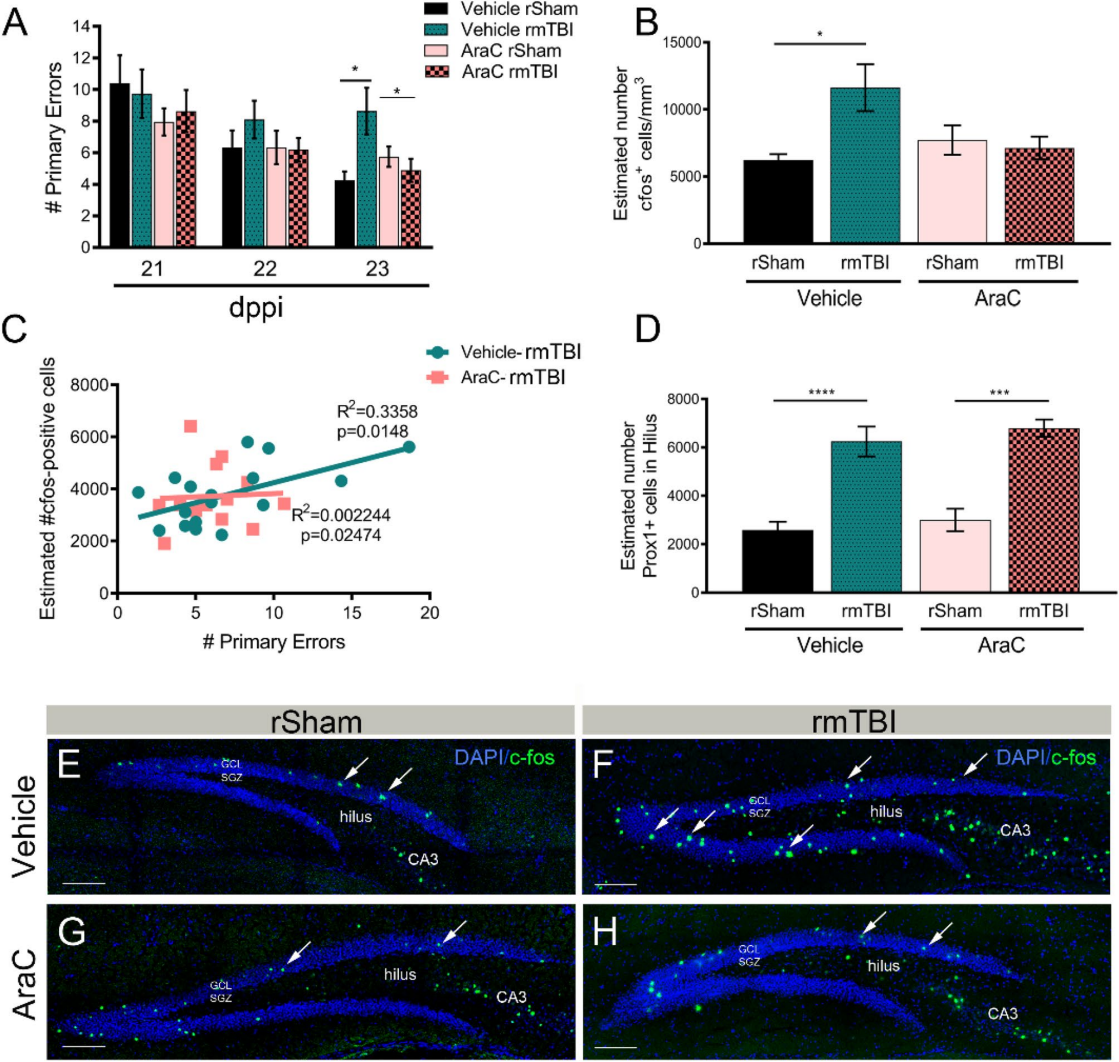
AraC recues cognitive deficits and hyperactivity in the DG following rmTBI. (**A**) The number of primary errors made by vehicle infused rmTBI mice was significantly reduced in rmTBI mice treated with AraC at 23 dppi. (**B**) Quantified data showing estimated number of cFos+ cells/mm^3^ by non-biased stereology in the DG at 24 dppi. Mice receiving pre-treatment with AraC did not display an increase in cFos activity in the DG after rmTBI compared to vehicle treated mice. (**C**) The Correlation Coefficient plotted for each animal, shows the number of cFos+ cells in the DG is positively correlated with the number of Barnes Maze errors (R^2^ = 0.3; *p* = 0.01) in vehicle-treated mice. No correlation was observed in AraC mice (R^2^ = 0.0; *p* = 0.02). (**D**) The number of Prox1+ cells quantified in the hilus was increased following 24 dppi rmTBI in vehicle but not AraC mice. (**E**–**H**) Representative confocal images of cFos (green) and DAPI (blue) staining in the DG of rSham and rmTBI mice pre-infused with vehicle or AraC. Scale = 500 µm in (**E**–**H**). *p < 0.05; ***p = 0.001; ****p = 0.0001. n = 9–13 mice per group for behavior and 6–10 for stereology.

Next, we tested whether abrogation of aberrant neurogenesis could alleviate learning and memory impairments in mice subjected to rmTBI. Using Barnes Maze, we found vehicle-treated rmTBI mice display a significant increase in the number of primary errors at 23 dppi compared to vehicle-treated rSham (Fig. 3A). Interestingly, we observed that Ara-C treated mice attenuated this effect (F_(3,37)_ = 3.33; *p* = 0.02), suggesting atypical neurogenesis may play an essential role in rmTBI-induced cognitive decline. No difference was observed in latency (F_(3,40)_ = 0.77; *p* = 0.51), or distance traveled (F_(3,38)_ = 1.14; *p* = 0.34) between groups. We then quantified the number of cFos-immunolabeled cells in the DG, as a marker of neuronal and functional activity in the brain^48^. Vehicle-treated rmTBI mice displayed a significant increase in the number of cFos-positive cells in the DG (Fig. 3B,F) compared to vehicle-treated rSham (Fig. 3B,E) that was not present in AraC-treated mice (Fig. 3B,G,H) (F_(3,25)_ = 3.39; *p* = 0.03). We further observed a direct correlation between the number of errors on the Barnes Maze and the estimated number of cFos-positive cells in the DG of vehicle-treated rmTBI mice (F_(1,16)_ = 48.02; *p* < 0.0001) that was not present in AraC-treated mice (Fig. 3C) (F_(1,11)_ = 0.76; *p* = 0.40). Aberrant migration of hilar ectopic granular cells have been suggested to contribute to hyperactivity in the hippocampus^49^. While we observed a significant increase of Prox1-positive cells in the dentate hilus of vehicle-treated rmTBI mice, this effect was not influenced by AraC treatment (Fig. 3D) (F_(3,26)_ = 17.37; *p* < 0.0001). Taken together, these data imply that the increased neurogenic response and hyperactivity in the DG contribute to learning and memory deficits following rmTBI.

### Vascular-specific EphA4 mediates aberrant neurogenesis and cognitive deficits following rmTBI

**Figure 4:**
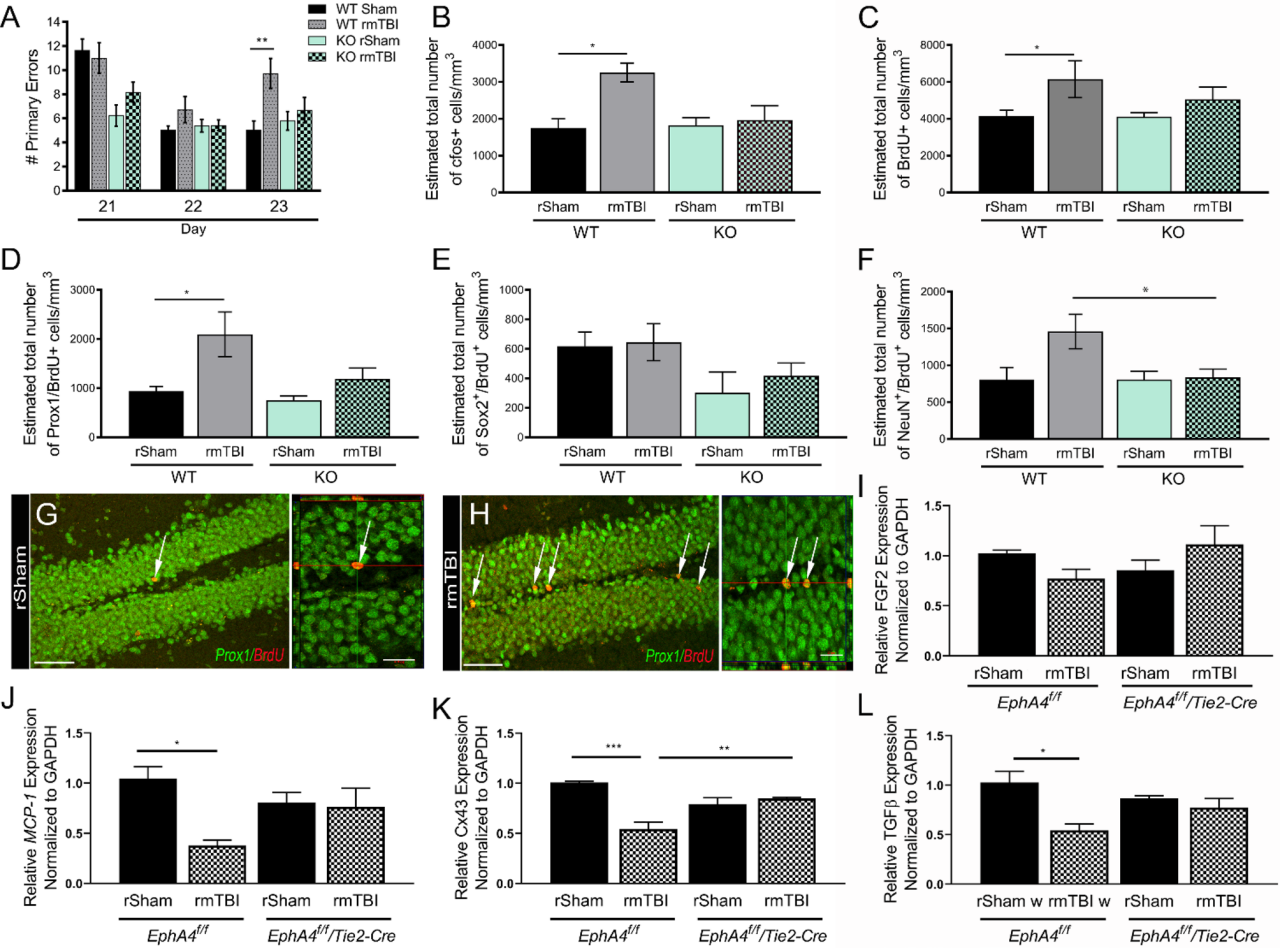
*EphA4f.*^*/f*^*/Tie2-Cre* mice show attenuation of rmTBI-induced deficits. (**A**) The number of primary errors in significantly increased in rmTBI EphA4f.^/f^ (WT) mice at 23 dppi compared rSham. This effect was not observed in EphA4f.^/f^/Tie2-Cre **(**KO) mice. Compared to rSham, rmTBI KO mice did not show an increase in the number of cFos (**B**), BrdU (**C**) or Prox1/BrdU (**D**) positive cells in the DG as was seen in WT mice. No difference in the number of Sox2/BrdU (**E**) or NeuN/BrdU (**F**) positive cells were found across the groups of mice. (**G**–**H**) Representative confocal images of Prox1/BrdU positive cells in the DG at 24 dppi in WT mice. (**I**–**L**) Quantified mRNA expression of total DG tissue using qPCR for *FGF2*, *MCP-1*, *Gja1 (Cx43)*, and *TGFβ*, respectively. All genes were normalized to GAPDH then represented relative to WT rSham levels. *p < 0.05; **p < 0.01, ***p = 0.001. Scale = 100 µm in (**G**,**H**); 20 µm in inset. n = 5–10 mice per group for stereology and n = 10–16 for behavior. qPCR was performed using biological triplicates.

Previous findings suggest that vascular distribution and activation of ephrin type-A receptor 4 (EphA4) influences hippocampal neurogenesis^30,31^. Given its emerging significance in vascular function^32–34^, we sought to determine the role of EphA4 in rmTBI-mediated effects using endothelial cell (EC)-specific EphA4 knockout (KO) mice (EphA4f.^/f^/Tie2-Cre) and wild type (WT) mice (EphA4f.^/f^)^32,33^. The experimental timeline was performed as in Fig. 1G. Barnes Maze analysis showed rmTBI results in significant learning and memory deficits in WT mice, which was not observed in KO mice at 23 dppi (Fig. 4A) (F_(3,46)_ = 4.67; *p* = 0.006). No difference was observed in latency (F_(3,38)_ = 2.0; *p* = 0.12) between groups. These findings correlated with an increase in the number of cFos+ (Fig. 4B) (F_(3,25)_ = 3.38; *p* = 0.03), BrdU+ (Fig. 4C) (F_(3,24)_ = 2.65; *p* = 0.07), and Prox-1+/BrdU+ (Fig. 4D,G,H) (F_(3,30)_ = 4.94; *p* = 0.006), cells in the DG of rmTBI compared to rSham in WT but not KO mice. No changes were found in the number of Sox2+/BrdU+ (Fig. 4E) (F_(3,21)_ = 1.73; *p* = 0.19), however, the number of NeuN+/BrdU+ (Fig. 4F) positive cells were found significant only between rmTBI groups (F_(3,28)_ = 4.037; *p* = 0.01). Finally, mRNA expression in the DG of WT and KO mice was assessed using quantitative real-time PCR (qPCR) for several genes well-known to be involved in neurogenesis^50–53^. DG tissue was microdissected at 24 dppi from rSham and rmTBI mice. While no difference was found in *FGF2* expression (Fig. 4I) (F_(3,8)_ = 1.75; *p* = 0.23), a significant reduction in *MCP-1* (Fig. 4J) (F_(3,8)_ = 5.03; *p* = 0.03), *Cx43* (Fig. 4K) (F_(3,8)_ = 15.75; *p* = 0.001), and *TGFβ* (Fig. 4L) (F_(3,8)_ = 6.32; *p* = 0.01), was observed in WT rmTBI mice compared to WT rSham. This downregulation, however, was not found in KO mice following rmTBI. These findings indicate EC-specific EphA4 contributes to the cognitive behavioral and hippocampal neurogenic changes induced by rmTBI.

## Discussion

Long-term cognitive impairments can result from repeated traumatic insult to the brain^54^. Our findings demonstrate, using a murine model of rmTBI^26^, that learning and memory deficits occur as early as 24 days post-initial injury. This neurological change coincides with an increased presence of BrdU-labeled Prox1+ neuroblasts or immature neurons in the DG that is not seen at 9 dppi. The loss of BrdU-labeled cells originating from the NSPC pool overtime is a normal process of neurogenesis. However, the sustained presence of BrdU/Prox1-positive cells in the DG at 24 dppi may result from enhanced proliferation and/or survival of the neuroblast population. This is evident due to the fact that the resulting pool of fate-labeled Sox2+ NSPCs and NeuN+ mature neurons were unchanged following rmTBI suggesting the immature neuron population is selectively affected by rmTBI. Transient suppression of neurogenesis, using AraC infusion to ablate dividing NSPCs prior to rmTBI, prevented the expansion of the Prox-1+ cell population at 24 dppi. This coincided with restoration of proper hippocampal-dependent learning and memory function and attenuation of enhanced cFos immunolabeling in the DG that represents neuronal hyperactivity^55^. While the expression of immediate early gene, cFos, is associated with learning and memory^48^, atypical expression can be found in areas of the brain that are hyperexcited or following seizure induction such as the DG, cortex, hippocampus and limbic system^56^. Changes in cFos have also been observed in the brain following stress or injury^57^. Here we show increased expression in the DG in response to rmTBI is positively correlated with behavioral deficits. Moreover, ectopic hilar migration of Prox1-positive cells influences DG function and has been observed following FPI model of TBI^15,58^. We also show ectopic migration of Prox1 granular neurons into the hilus following rmTBI was not prevented by AraC treatment, suggesting this occurrence may not contribute to memory impairments assessed by Barnes Maze testing. Our findings further validate the negative role of aberrant hippocampal neurogenesis in functional cognitive outcomes as a result of repeated mild trauma to the brain.

Vascular involvement in hippocampal neurogenesis has been postulated to regulate neural progenitor cell behavior and responses to external stimuli^59–61^. Blood vessels act as physical substrates for proliferating neuroblasts during tangential migration in the DG^62^. Vascular-neuroblast interactions are a crucial niche component necessary for cell division and migration status of immature neurons^62–64^. Vascular endothelial cell (EC) expression of EphA4 receptor tyrosine kinase has been implicated in regulating neurogenesis in the DG^31^. Cell-to-cell contact involving membrane bound ephrin ligand and Eph receptor signaling plays a key role in neurogenesis^39,65,66^. We previously generated cell-specific EphA4 KO mice using the Tie2 promoter (EphA4f.^/f^/Tie2-Cre), which results in deletion of EphA4 in ECS and hematopoietic cells^32,33^. While loss of EC-specific EphA4 does not result in observable changes in neurogenesis using fate labeling in un-injured mice, we find that rmTBI-induced increase in BrdU/Prox1 double-positive cell population was attenuated in KO mice. This correlated with a rescue of learning and memory deficits and neuronal activity as quantified by cFos expression in the DG. These findings suggest that vascular expression and function of EphA4 mediates overproduction of immature neurons in the DG following rmTBI. Given the loss of EphA4 in hematopoietic cells in our KO mice, we cannot rule out the possibility of peripheral immune cell-specific contributions which will require further investigation. However, the BBB has not been shown to be disrupted in this model.

Lastly, rmTBI results in chronic downregulation of *MCP-1*, *Gja1 (Cx43)* and *TGFβ* mRNA expression in the microdissected DG of WT mice, which was not seen in KO mice. Importantly, rmTBI KO mice show a statistically significant attenuation in Cx43 downregulation compared to WT rmTBI suggesting Cx43 may mediate EC-specific EphA4 effects following injury. Previous studies show hippocampal multipotent progenitor cells display reduced levels of *Cx43* during intermediate stage of neuronal differentiation^67^. This may reflect the change in DG composition which shows increased immature neuron production after rmTBI. Studies also show using single cell RNA sequencing of the mouse DG, that Cx43 is downregulated in proliferating neural progenitor cells^68^. While the expression of EC-specific Cx43 in the DG has not been investigated, the role of vascular influence in hippocampal neurogenesis through gap junction (GJ)-associated Cx43 remains to be established. Interestingly, our unpublished findings show enhanced expression of Cx3 protein in cultured EphA4-null ECs compared to WT ECs (data not shown). Further studies are needed to determine whether EC-specific EphA4 activation may mediate expansion of the neuroblast or immature neuron population by suppressing Cx43 expression and/or function during neuroblast-vascular coupling.

While the controversial role of aberrant neurogenesis in the pathogenesis of TBI remains under investigation, our findings highlight a negative role in cognitive outcomes in a murine model of repeated mild injury. Through fate labeling, we show that the immature neuron population is selectively influenced by rmTBI and that vascular expression of EphA4 may mediate this phenotype by regulating Cx43 expression in the DG. Overproduction of immature neurons correlates with hyperactivity as seen through enhanced presence of cFos DG staining. This effect directly coincided with the number of errors made by rmTBI mice on the Barnes Maze spatial learning task which was rescued following neurogenic suppression using AraC. This implies that aberrant neurogenesis may interrupt the normal hippocampal circuitry required for proper learning and memory tasks. Findings from this study uncover a novel mechanism regulating the atypical neurogenic response to rmTBI which may be exploited for future hypothesis-driven research.

**Table 1:** Information of antibodies.

Antibody	Provider	Catalog number	Dilution
BrdU	Abcam	Ab6326	1:500
Prox1	ECM biosciences	CM4961	1:500
Sox2	Cell signaling technology	3511S	1:250
GFAP	Cell signaling technology	D1F4Q	1:500
CD31	R&D Systems	AF3628	1:500
NeuN	Cell signaling technology	12943S	1:500
cFos	Cell signaling technology	2250	1:500

**Table 2:** qPCR primers.

Gene	Primer sequence (5′–3′)
*Gapdh*	Fw: CGTCCCGTAGACAAAATGGT Rv: TCAATGAAGGGGTCGTTGAT
*Gja-1 (Cx43)*	Fw: CGGAAGCACCATCTCCAACT Rv: CCACGATAGCTAAGGGCTGG
*FGF2*	Fw: GCCTTTGTGCCTTCCCTAGT Rv: GCCGGCCATGGAAAATTCTG
*Tgfβ*	Fw: TTAGGAAGGACCTGGGTTGGA Rv: CAGGGTCCCAGACAGAAGTT
*MCP1*	Fw: TCACCTGCTGCTACTCATTCACCA Rv: TACAGCTTCTTTGGGACACCTGCT

## Supplementary information

Supplementary Information.
