# Supplementary material for: Abrogation of atypical neurogenesis and vascular-derived EphA4 prevents repeated mild TBI-induced learning and memory impairments
Source: Sci Rep. 2020 Sep 21;10:15374. doi: 10.1038/s41598-020-72380-1 (PMC7506550; doi:10.1038/s41598-020-72380-1)
Supplement: Supplementary file 1 — Supplementary Information. [file 41598_2020_72380_MOESM1_ESM.docx]

**Abrogation of atypical neurogenesis and vascular-derived EphA4 prevents repeated mild TBI-induced learning and memory impairments**

Kisha Greer^1^, Erwin Kristobal Gudenschwager Basso^2^, Colin Kelly^2^, Alison Cash^2^, Elizabeth Kowalski^2^, Steven Cerna^2^, Collin Tanchanco Ocampo^2^, Xia Wang^2^ and Michelle H. Theus^1, 2, 3,4*^

^1^Graduate Program in Translational Biology, Medicine, and Health, Virginia Tech, Blacksburg, VA 24061, USA

^2^The Department of Biomedical Sciences and Pathobiology, Virginia Tech, Blacksburg, VA 24061, USA

^3^Center for Regenerative Medicine, Virginia-Maryland College of Veterinary Medicine, Blacksburg, Virginia, 24061, USA

^4^Center for Engineered Health, Virginia Tech, Blacksburg, Virginia, 24061, USA

^*^Corresponding Author

**Corresponding author:**

Michelle Theus, PhD

Co-Director Translational Biology Medicine and Health graduate program

Associate Professor, Biomedical Sciences and Pathobiology, Virginia Tech

Associate Professor, Health Sciences, Virginia Tech

Affiliate, VT-Biomedical Engineering and School of Neuroscience

970 Washington Street SW

Life Sciences I; rm 249 (MC0910)

Blacksburg, VA 24061

Tel. 540-231-0909; Fax 540-231-7425; E-mail: [**mtheus@vt.edu**](mailto:mtheus@vt.edu)


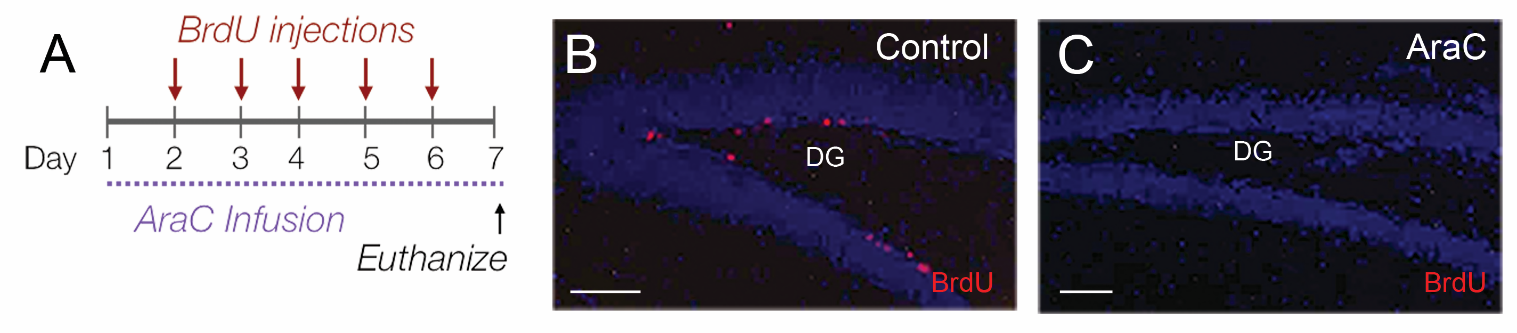


**Supplemental Fig. 1. (A)** Experimental design to test the effects of 2% AraC infusion or vehicle control on proliferation in the DG using 50mg/kg/day BrdU injections in naïve mice. At 7 days post-infusion, BrdU labeling in the subgranular zone (SGZ) of vehicle control mice shows numerous BrdU-labeled cells in the SGZ **(B)**. AraC ablation suppressed the number of BrdU-positive cells in the DG **(C)**. Scale = 500µm in B and C.
